# Supplementary material for: A non-cell-autonomous role for Pml in the maintenance of leukemia from the niche
Source: Nat Commun. 2018 Jan 4;9:66. doi: 10.1038/s41467-017-02427-x (PMC5754357; doi:10.1038/s41467-017-02427-x)
Supplement: Supplementary file 1 — Supplementary Information [file 41467_2017_2427_MOESM1_ESM.pdf]

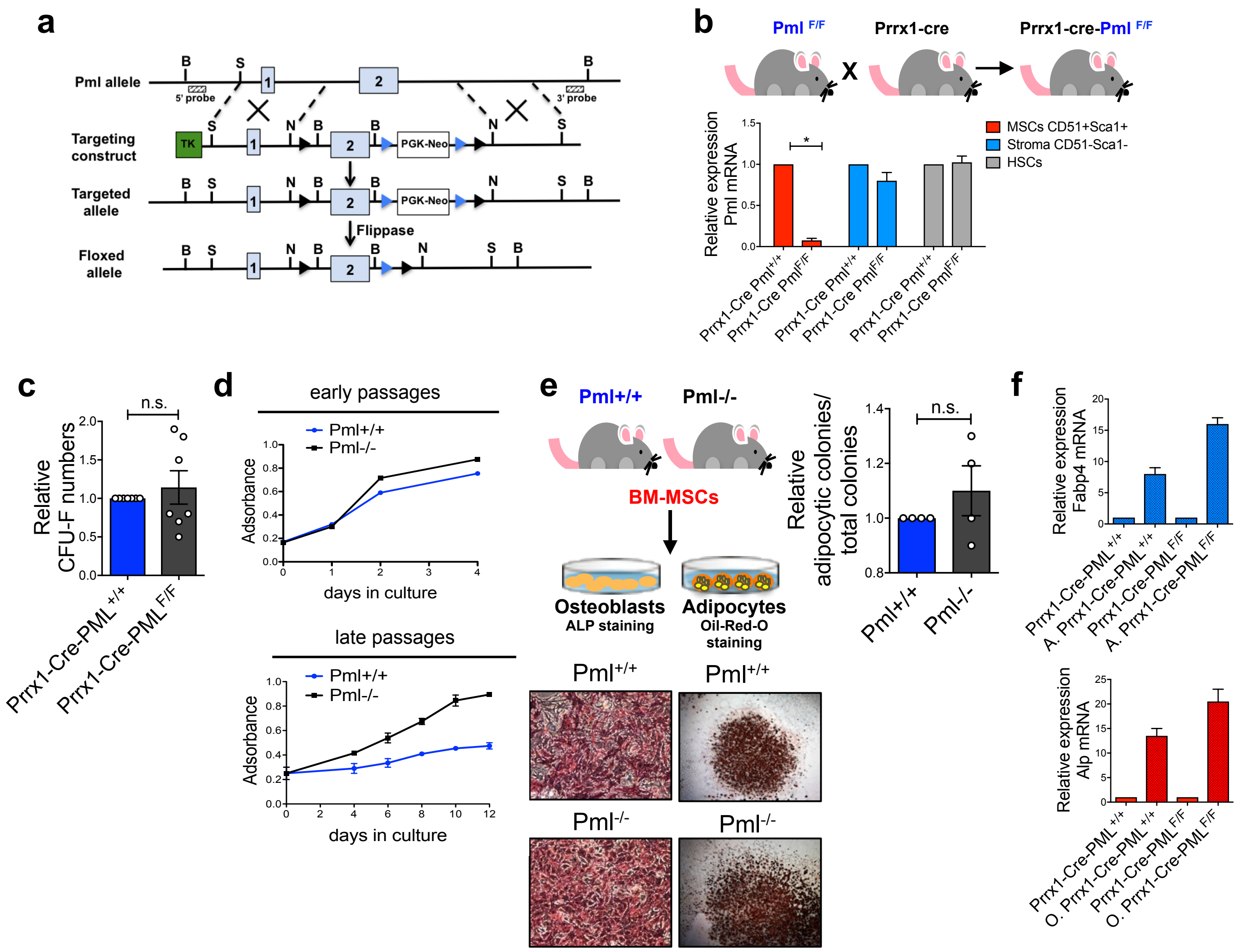

Supplementary Figure 1 | Pml regulates the biology of MSCs. (**a**) Schematic overview of the experimental design used to generate *Pml-floxed* mice. (**b**) Expression levels, assayed by RT-qPCR, of Pml mRNA in the different populations of mesenchymal cells of *Prrx1-Cre-Pml*$^{+/+}$ or *Prrx1-Cre-Pml*$^{F/F}$ mice; HSCs are used as control. Cells collected from n=3 mice for each genotype were pooled together prior to RNA extraction. (**c**) CFU-F colonies forming the capacity of MSCs derived from *Prrx1-Cre-Pml*$^{F/F}$ or Prrx1-Cre-Pml$^{+/+}$ mice (n=7). (**d**) Proliferation of MSCs derived from *Pml*$^{+/+}$ or *Pml*$^{-/-}$ mice either at early passages *in vitro*, or at late passages. One representative experiment is shown. (**e**) Differentiation of MSCs derived from *Pml*$^{+/+}$ or *Pml*$^{-/-}$ mice into osteoblasts or adipocytes. Osteoblasts (on the left) were stained with Alkalyne Phosphatase (ALP), while adipocytes (on the right) were stained Oil-Red-O. The chart on the right shows the quantification of Oil-Red-O positive colonies (n=4). (**f**) Capacity of *Prrx1-Cre-Pml*$^{+/+}$ or *Prrx1-Cre-Pml*$^{F/F}$ MSCs to differentiate to adipocytes (upper chart, A= MSCs triggered to adipogenesis), or into osteoblasts (lower chart. O= MSCs triggered to osteogenesis) once triggered with specific factors. The chart on the upper right panel shows the relative expression of Fabp4, while the one on the bottom shows the expression of Alp. One representative experiment ± SEM is shown.

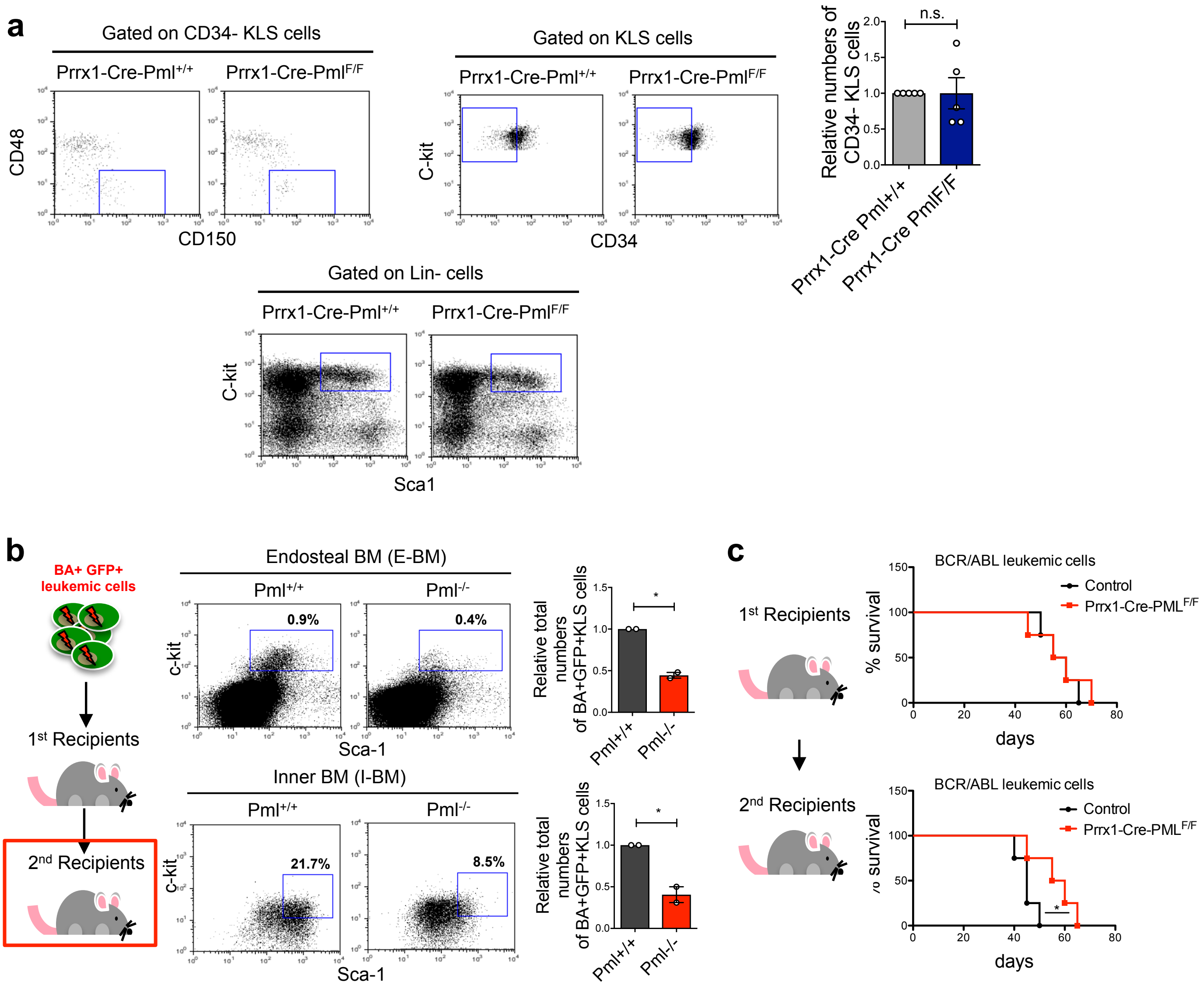

Supplementary Figure 2 | Pml expressed in MSCs regulates only marginally non-cell-autonomously HSCs. (**a**) Representative plots showing the hematopoietic sub-populations in *Prrx1-Cre-Pml*$^{+/+}$ or *Prrx1-Cre-Pml*$^{F/F}$ mice. The upper left plot shows CD150+CD48-CD34-KLS cells; the one on the right shows CD34-KLS cells, while the one on the bottom shows KLS cells. The chart on the right shows the quantification of CD34-KLS cells in *Prrx1-Cre-Pml*$^{+/+}$ or *Prrx1-Cre-Pml*$^{F/F}$ mice (n=5 mice analyzed). (**b**) Relative percentages (representative plots on the left) and relative total numbers (charts on the right) of BA^{+}GFP^{+}KLS cells present at the endosteal bone marrow (E-BM), or within the inner cavity of the bone marrow (I-BM) in second recipients *Pml*$^{+/+}$ or *Pml*$^{-/-}$ (n=2 mice analyzed). (**c**) Survival curves of serially transplanted *Prrx1-Cre-Pml*$^{F/F}$ or control mice with BA+GFP + cells. The upper panel shows the survival of primary recipients, while the bottom panel shows the survival of secondary recipient mice.

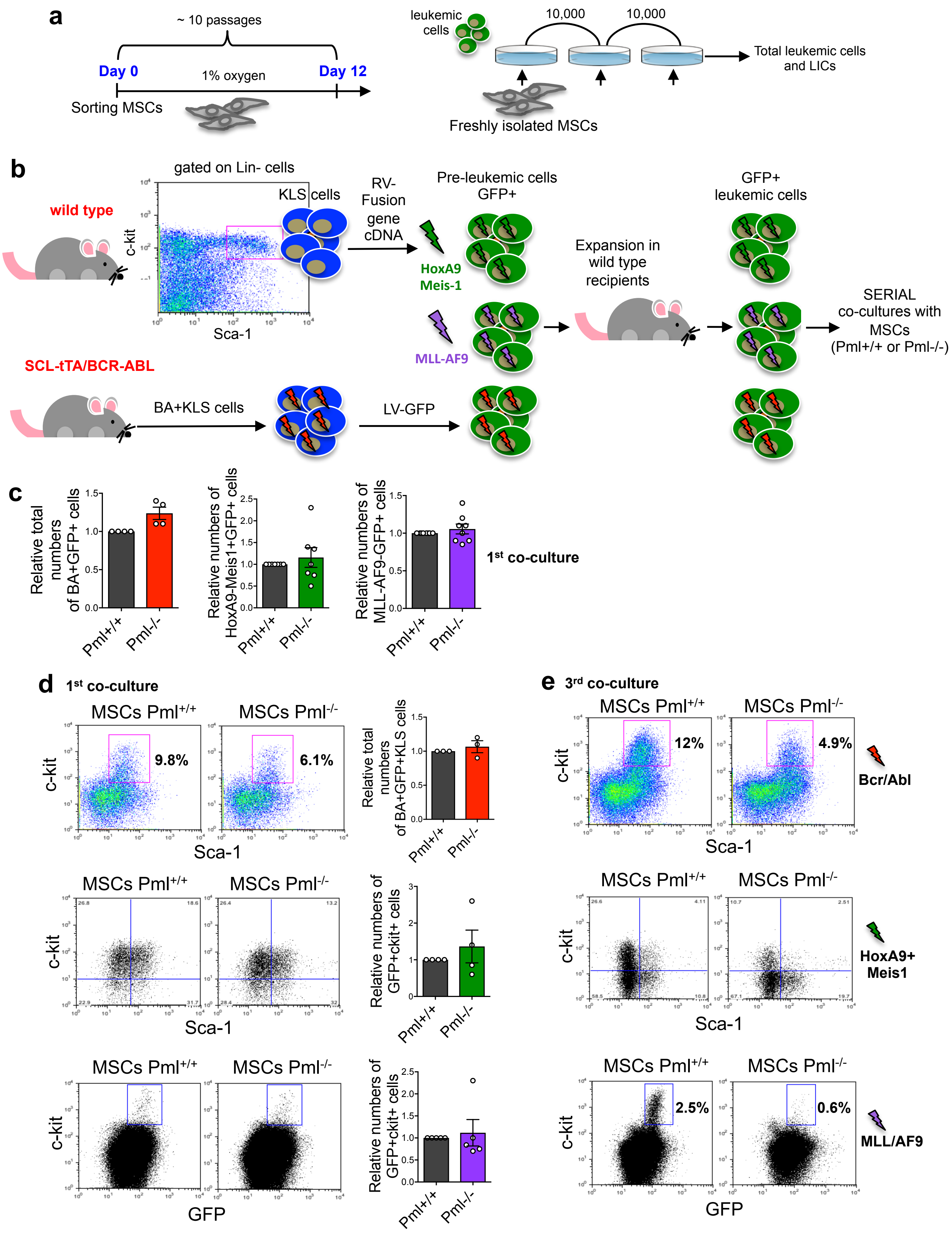
a
~ 10 passages
Day 0
1% oxygen
Day 12
Sorting MSCs
leukemic cells
10,000
10,000
Total leukemic cells and LICs
Freshly isolated MSCs
b
gated on Lin- cells
KLS cells
wild type
c-kit
Sca-1
RV-Fusion gene cDNA
Pre-leukemic cells GFP+
HoxA9 Meis-1
MLL-AF9
Expansion in wild type recipients
GFP+ leukemic cells
SERIAL co-cultures with MSCs (Pml+/+ or Pml-/-)
SCL-tTA/BCR-ABL
BA+KLS cells
LV-GFP
c
Relative total numbers of BA+GFP+ cells
Relative numbers of HoxA9-Meis1+GFP+ cells
Relative numbers of MLL-AF9-GFP+ cells
Pml+/+
Pml-/-
1st co-culture
d
1st co-culture
MSCs Pml+/+
MSCs Pml-/-
9.8%
6.1%
Relative total numbers of BA+GFP+KLS cells
Relative numbers of GFP+ckit+ cells
GFP
e
3rd co-culture
12%
4.9%
Bcr/Abl
HoxA9+ Meis1
2.5%
0.6%
MLL/AF9

Supplementary Figure 3 | Pml expressed in MSCs regulates non-cell-autonomously LICs. (**a**) Schematic overview of MSCs culturing conditions, and of the co-cultures with MSCs and leukemic cells. (**b**) Schematic overview of the experimental design to generate leukemic cells that express HoxA9-Meis1, MLL/AF9, or GFP+BCR/ABL. (**c**) Relative numbers of BA+GFP+ cells, HoxA9+Meis1 leukemic cells and MLL/AF9 leukemic cells derived from the first co-cultures with MSCs $Pml^{+/+}$ or $Pml^{-/-}$ (n≥4). (**d**) Relative numbers and percentages of BA+GFP+KLS cells, HoxA9-Meis1 GFP+c-kit+ cells, and MLL/AF9 GFP+c-kit+ cells in the first co-cultures with $Pml^{+/+}$ or $Pml^{-/-}$ MSCs. Quantifications are represented in the charts on the right, while representative plots are shown on the left (n≥3). (**e**) Representative plots showing percentages of BA+GFP+KLS cells, HoxA9-Meis1 GFP+c-kit+ cells, and MLL/AF9 GFP+c-kit+ cells in serial co-cultures with $Pml^{+/+}$ or $Pml^{-/-}$ MSCs.

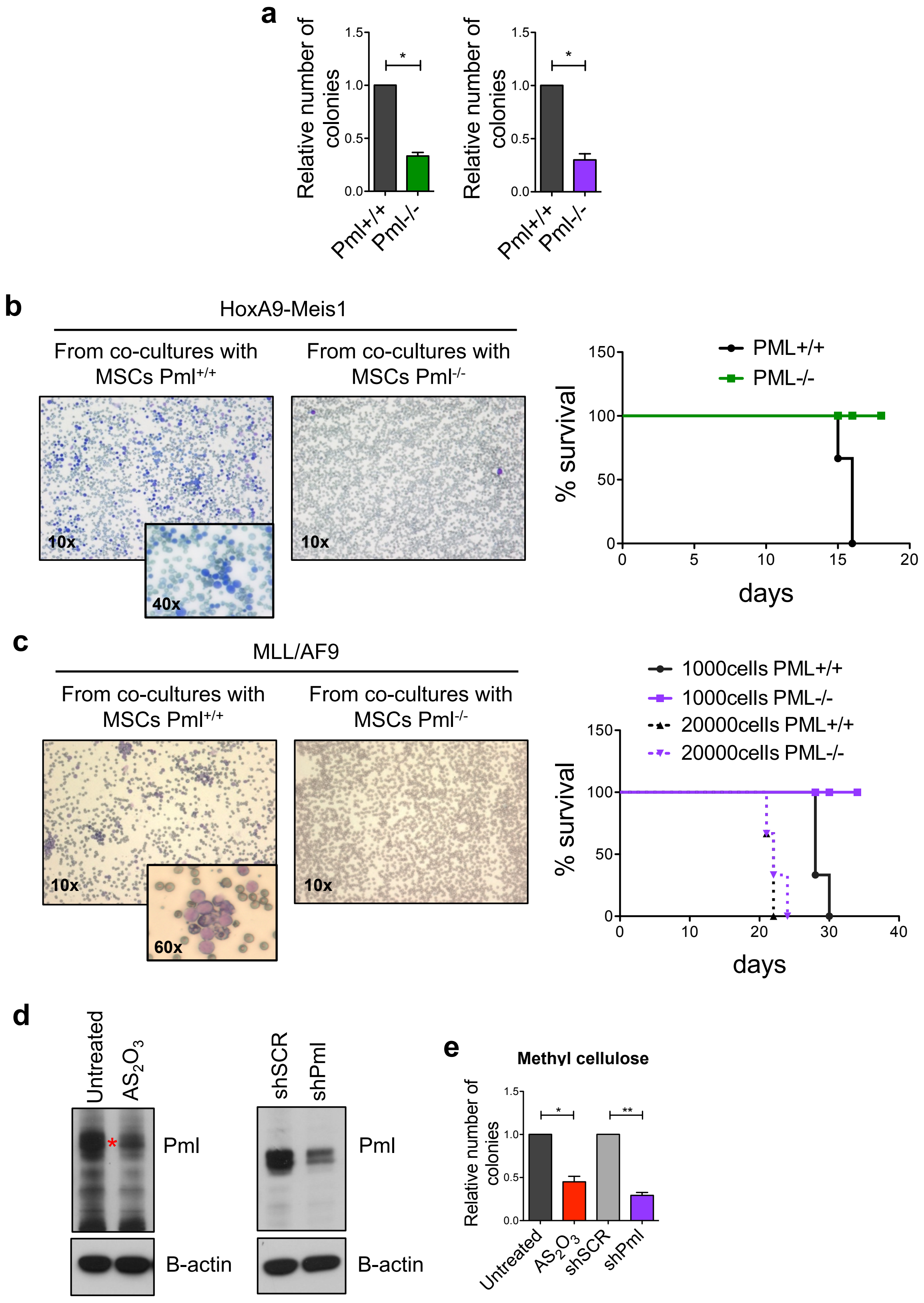
a
Relative number of colonies
1.5
1.0
0.5
0.0
*
Pml+/+
Pml-/-
Relative number of colonies
1.5
1.0
0.5
0.0
*
Pml+/+
Pml-/-
b
HoxA9-Meis1
From co-cultures with MSCs Pml+/+
From co-cultures with MSCs Pml-/-
10x
40x
10x
PML+/+
PML-/-
150
100
50
0
% survival
0
5
10
15
20
days
c
MLL/AF9
From co-cultures with MSCs Pml+/+
From co-cultures with MSCs Pml-/-
10x
60x
10x
1000cells PML+/+
1000cells PML-/-
20000cells PML+/+
20000cells PML-/-
150
100
50
0
% survival
0
10
20
30
40
days
d
Untreated
AS2O3
Pml
B-actin
shSCR
shPml
Pml
B-actin
e
Methyl cellulose
Relative number of colonies
1.5
1.0
0.5
0.0
*
**
Untreated
AS2O3
shSCR
shPml

Supplementary Figure 4 | Pml expressed in MSCs regulates leukemic cells in a non-cell-autonomous manner. (**a**) Relative number of colonies in methyl-cellulose, generated by leukemic cells derived from co-cultures with MSCs *Pml*$^{+/+}$ or *Pml*$^{-/-}$. HoxA9-Meis1 leukemic cells are shown on the left, while MLL/AF9 leukemic cells are shown on the right. One experiment ± SEM is shown. (**b**) H&E of leukemic blasts in the blood of mice transplanted with HoxA9-Meis1 leukemic cells after co-culture with *Pml+/+* or *Pml-/-* MSCs. The survival curve of these mice is shown on the right. (**c**) H&E of leukemic blasts in the blood of mice transplanted with MLL/AF9 leukemic cells after co-culture with *Pml+/+* or *Pml-/-* MSCs. The survival curve of these mice is shown on the right. (**d**) Western blot analysis of Pml expression in MSCs upon knock-down with shRNA and upon treatment with AS_2O_3. (**e**) Methyl-cellulose assay performed with leukemic cells (MLL/AF9) which have been co-cultured with MSCs, while treating with AS_2O_3, or performed with leukemic cells co-cultured with MSCs transduced with an shRNA targeting Pml. One experiment ± SEM is shown.

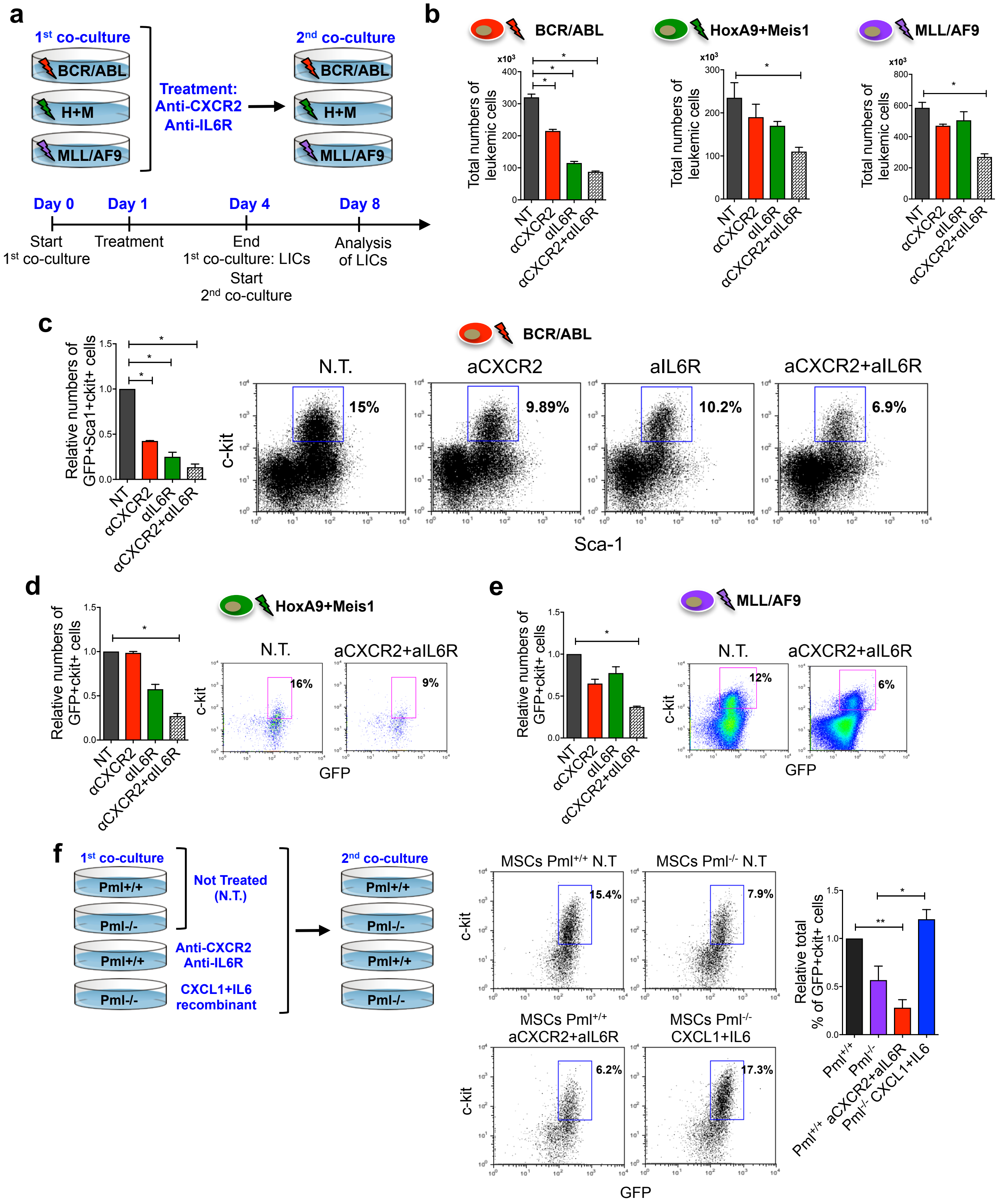

a
1st co-culture
BCR/ABL
H+M
MLL/AF9
Treatment:
Anti-CXCR2
Anti-IL6R
2nd co-culture
BCR/ABL
H+M
MLL/AF9
Day 0
Day 1
Day 4
Day 8
Start
1st co-culture
Treatment
End
1st co-culture: LICs
Start
2nd co-culture
Analysis
of LICs
b
BCR/ABL
HoxA9+Meis1
MLL/AF9
Total numbers of
leukemic cells
NT
αCXCR2
αIL6R
αCXCR2+αIL6R
c
BCR/ABL
Relative numbers of
GFP+Sca1+ckit+ cells
N.T.
aCXCR2
aIL6R
aCXCR2+aIL6R
15%
9.89%
10.2%
6.9%
c-kit
Sca-1
d
HoxA9+Meis1
Relative numbers of
GFP+ckit+ cells
N.T.
aCXCR2+aIL6R
16%
9%
c-kit
GFP
e
MLL/AF9
Relative numbers of
GFP+ckit+ cells
N.T.
aCXCR2+aIL6R
12%
6%
c-kit
GFP
f
1st co-culture
Pml+/+
Pml-/-
Not Treated
(N.T.)
Anti-CXCR2
Anti-IL6R
CXCL1+IL6
recombinant
2nd co-culture
MSCs Pml+/+ N.T
MSCs Pml-/- N.T
MSCs Pml+/+
aCXCR2+aIL6R
MSCs Pml-/-
CXCL1+IL6
15.4%
7.9%
6.2%
17.3%
c-kit
GFP
Relative total
% of GFP+ckit+ cells
Pml+/+
Pml-/-
Pml+/+ aCXCR2+aIL6R
Pml-/- CXCL1+IL6

Supplementary Figure 5 | Pml regulates leukemic cells non-cell-autonomously through IL6/IL6R and CXCL1/CXCR2 pathways. (**a**) Schematic overview of the experimental design for treatment of the co-cultures with anti-IL6R and anti-CXCR2 antibodies. (**b**) Total number of leukemic cells not treated (NT) or treated with anti-Il6R, anti-CXCR2 singularly or in combination. One representative experiment ± SEM is shown. (**c**) Total numbers of BA+GFP+KLS cells after the treatment with anti-IL6R or/and anti-CXCR2, singularly or in combination. Not treated cells (NT) were used as control. The chart on the left shows the quantification, while representative plots are shown on the right. One representative experiment ± SEM is shown. (**d**) Chart on the left shows the numbers of HoxA9-Meis1+GFP+ckit+ cells after the first co-culture and treatment with anti-IL6R or/and anti-CXCR2 antibodies (singularly or in combination) One representative experiment ± SEM is shown; representative plots are shown on the right. (**e**) Chart on the left shows the numbers of MLL-AF9+GFP +ckit+ cells after the first co-culture and treatment with anti-IL6R or/and anti-CXCR2 antibodies (singularly or in combination) One representative experiment ± SEM is shown; representative plots are shown on the right. (**f**) Schematic overview of the experimental design is shown on the left. Co-cultures of MSCs $Pml^{+/+}$ and HoxA9-Meis1^{+}GFP^{+} leukemic cells were untreated (NT) as control, or treated with anti-CXCR2 in combination of anti-IL6R. Co-cultures with MSCs $Pml^{-/-}$ and HoxA9-Meis1^{+}GFP^{+} leukemic cells were untreated as control, or treated with recombinant Il6 and Cxcl1 proteins. Leukemic cells were then re-plated onto the new MSCs ($Pml^{+/+}$ or $Pml^{-/-}$); secondary co-cultures were analyzed. Percentages of GFP+ckit+ cells in the different conditions are shown in the plots and the chart on the right. One experiment ± SEM is shown.

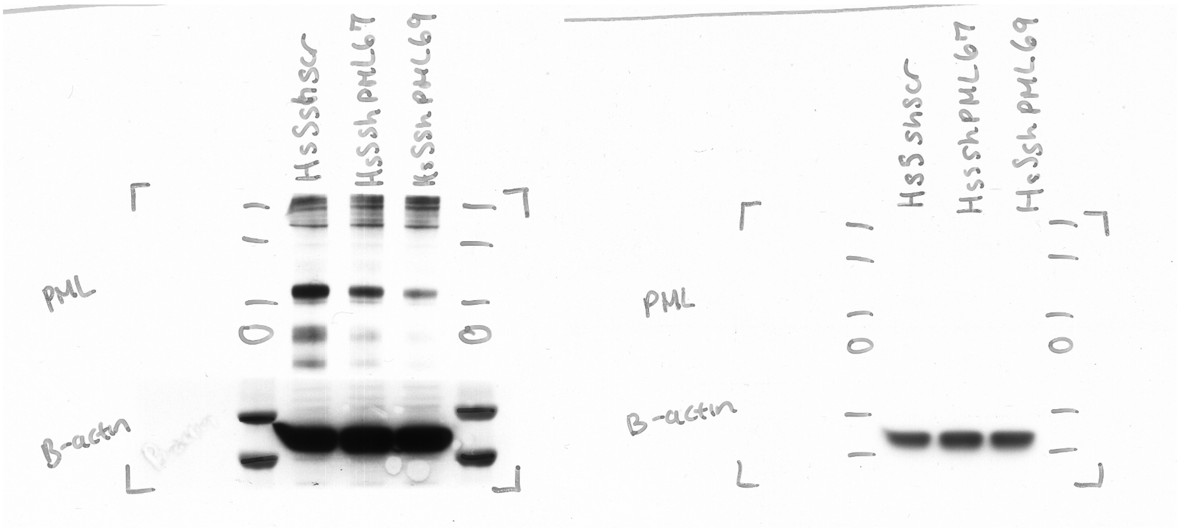

Supplementary Figure 6 | Original blots of Figure 5c
